# Factors Associated with the Health Status of Caregivers of Community-dwelling Older Adults with Dementia in Japan: The Toyama Dementia Survey

**DOI:** 10.31662/jmaj.2025-0508

**Published:** 2026-08-07

**Authors:** Nobue Nakahori, Masaaki Yamada, Michikazu Sekine, Takashi Tatsuse

**Affiliations:** 1 Department of Gerontological Nursing, School of Medicine, University of Toyama, Toyama, Japan; 2 Department of Epidemiology and Health Policy, School of Medicine, University of Toyama, Toyama, Japan

**Keywords:** dementia, family caregivers, health problems, behavioral and psychological symptoms of dementia

## Abstract

**Introduction:**

Maintaining the health of caregivers providing home care for older adults with dementia is necessary. This study aimed to investigate the prevalence of physical and mental health problems among these caregivers and to identify the associated factors.

**Methods:**

The Toyama Dementia Survey, a county-wide cross-sectional study, was conducted in 2014. In the first stage, 0.5% of the total geriatric population (307,582 residents) in Toyama prefecture was randomly sampled, and 1,303 individuals who agreed to participate were selected. They were screened via face-to-face interviews, including memory tests. Among them, 252 (19.3%) were identified as having a high probability of dementia. In the second stage, home visits were conducted by psychiatrists and public health nurses. While psychiatrists diagnosed care recipients with dementia, public health nurses interviewed caregivers about their health problems. The analysis focused on 75 pairs of care recipients and their family caregivers who were visited. The association between behavioral and psychological symptoms of dementia (BPSDs) and the physical and mental health of family caregivers was examined using logistic regression analysis.

**Results:**

Physical and mental health problems were experienced by 40% and 37.3% of the caregivers, respectively. The adjusted odds ratio (OR) for physical health problems among caregivers aged 80 years or older compared with those under 60 years was 13.46 (95% confidence interval [CI] 1.86-7.55). Compared with caregivers with patients without BPSDs, caregivers of patients with two or more BPSDs showed a marginal association with mental health problems (adjusted OR 2.96, 95% CI 0.84-10.43). Among physical symptoms, sleep deprivation was significantly more common in male caregivers than in female caregivers.

**Conclusions:**

Approximately 40% of caregivers of older adults with dementia experienced physical or mental health problems. Caregivers’ age was associated with physical health problems. Male caregivers were more likely to experience sleep deprivation than female caregivers.

## Introduction

Globally, dementia affects more than 55 million people, with caregivers such as family members and close friends providing an average of 5 hours of care and supervision daily ^(1)^. In Japan, the aging population is rapidly increasing, with a ratio reaching 29.3% in 2024 ^(2)^. According to a nationwide survey, approximately 4.4 million people, equivalent to 15% of Japan’s total older adult population, have dementia ^(3)^. With the various dementia policies implemented in Japan, the recent prevalence rate of dementia is lower than past estimates ^(4)^. However, Japan’s rapid aging and declining birth rate have led to labor shortages across all sectors. In home care, the number of family members who can provide care is decreasing. Family caregivers, such as older spouses or children in their prime working years caring for aging spouses or parents, are suffering from heavy burdens.

In 2000, the Japanese government introduced the Long-Term Care Insurance System to reduce family caregivers’ burden and provide various services to older adults requiring care ^(5)^. Thus, this system aids in alleviating the burden on family caregivers who provide care for older adults requiring care at home ^(6)^. However, home-based services available under this system have usage limits based on care level and are often provided on an hourly basis during the day. Consequently, the burden of providing continuous, 24-hour care, especially at night, falls on cohabiting family members. Furthermore, as cognitive function declines, care recipients may struggle to understand the need for medical visits or services. This difficulty can lead to refusal, potentially preventing caregivers from availing services. The burden on family caregivers reportedly remains high. They experience more health problems, including increased rates of depression, heart disease, and mortality, compared with noncaregivers ^(7), (8), (9)^.

Previous studies have identified factors related to caregiver burden and poor mental health. These include caregiver-related factors (e.g., age, sex, and income level) ^(10), (11)^ and care recipient–related factors (e.g., dementia severity, dementia type, and behavioral and psychological symptoms of dementia [BPSDs] frequency) ^(10), (15)^. Furthermore, caregivers’ burden and health status can be measured using scales such as the Zarit Burden Interview (ZBI; often referred to as the Zarit Burden of Care Inventory) ^(16), (17)^, the Neuropsychiatric Inventory Caregiver Distress Scale (NPI-D) ^(18)^, and the Short-Form Health Survey (SF-8) ^(19), (20)^ to determine their health conditions. However, identifying the factors associated with caregiver health problems and the relative strength of each factor’s influence is difficult for healthcare professionals and caregivers. Specifically defining caregiver health outcomes and analyzing them separately by dependent variables, such as physical and mental health problems, are necessary to clearly explain the impact of caregiver health problems on those involved in caregiving. Amid limited caregiving services, maintaining caregivers’ health while effectively using these services is a critical challenge for families caring for patients with dementia.

Most of the studies on patients with dementia have been conducted in clinical settings. Thus, these studies are susceptible to selection bias, and the prevalence rates of health problems among caregivers may not be generalizable. In Japan, where the demand for care for older adults with dementia is rapidly increasing, community-based studies investigating the health status of caregivers remain limited.

This study aimed to evaluate the prevalence of physical and mental health problems among family caregivers of community-dwelling older adults with dementia and to identify factors associated with the caregivers’ physical and mental health. Identifying these factors may help predict the occurrence of physical and mental health problems among family caregivers of older adults with dementia and facilitate appropriate interventions.

## Materials and Methods

### Study design and participants

The Toyama Prefecture Survey on the Status of Elderly Persons with Dementia is a prefecture-wide survey conducted in 1983, 1985, 1990, 1996, 2001, and 2014. Questions regarding caregiver health were first included in the 2014 survey; therefore, this study used data from that survey. A two-stage survey design was employed. In the first stage, out of 307,582 older residents of Toyama prefecture, 1,537 (0.5%) adults aged 65 years or older were randomly selected. Of these, 1,303 agreed to participate, yielding a response rate of 84.8% (1,303/1,537). These participants subsequently underwent face-to-face screening interviews conducted by public health nurses ^(21)^. During the interviews, questionnaires, including the Hasegawa Dementia Scale-Revised (HDS-R), were administered. The HDS-R is a reliable memory test widely used for screening dementia in Japan ^(22)^. A total of 252 older adults (19.3%) were identified as having suspected dementia and were included in the second stage. Among them, 180 older adults (71.4%) consented to receive home visits from designated psychiatrists and public health nurses. Before the survey, the psychiatrists and public health nurses received thorough training based on a screening manual. The selection criteria for caregivers were as follows: (1) living with and providing care for older adults and (2) the older adults being diagnosed with dementia according to the International Classification of Diseases, 10th Revision ^(23)^ based on the physical examination and Mini-Mental State Examination results ^(24)^. Older adults who did not meet the dementia criteria, such as those with depression, schizophrenia, or mild cognitive impairment, were excluded.

The flow of participant selection is shown in Figure 1.

**Figure 1.**
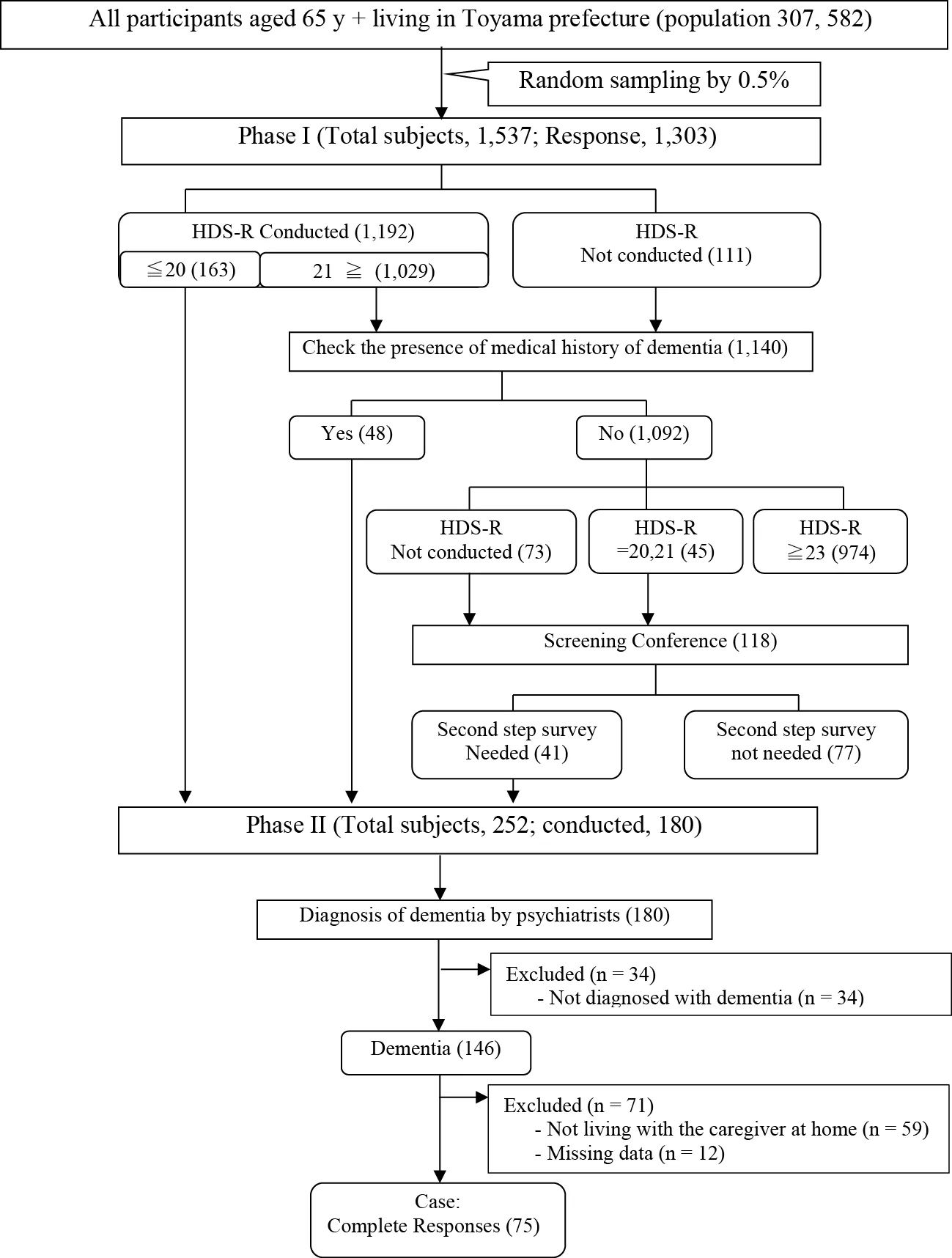
Flowchart of the Toyama Dementia Survey.

### Comprehensive assessment of dementia in care recipients and health problems in caregivers

A psychiatrist conducted physical and neurological examinations of care recipients to determine whether they met the criteria for dementia and to assess the type of dementia. Subsequently, a public health nurse interviewed the caregivers regarding their care recipients’ BPSDs, which were identified using an original checklist. To align with the widely used NPI-D assessment scale ^(18)^, this analysis focused on eight symptoms: delusions, hallucinations, agitation, depression, anxiety, apathy, irritability, and abnormal behavior. Additionally, caregivers were interviewed to assess their physical and mental health problems. Physical health problems included lower back pain, joint pain in the hands and feet, insufficient sleep, fatigue, and other symptoms, while mental health problems included irritability, depression, insomnia, and other symptoms.

### Variables

Exposure: The type of dementia in care recipients (Alzheimer’s disease, vascular dementia, mixed dementia, Lewy body dementia, and other dementia types), the number of BPSDs in care recipients (categorized as 0, 1, and ≥2), availability of a caregiver substitute (yes/no), and use of the Long-Term Care Insurance System (yes/no).

Outcome: The presence of physical or mental health problems among caregivers (physical health problems were defined as having at least one symptom among back pain, joint pain in the extremities, sleep deprivation, shoulder stiffness, fatigue, and other symptoms; mental health problems were defined as having at least one symptom among irritability, insomnia, depressive symptoms, and other symptoms).

Covariates: Caregiver age (<60, 60-69, 70-79, and ≥80 years) and caregiver sex (male, female).

### Statistical analysis

Regarding participant characteristics, we calculated the proportions of caregivers experiencing at least one physical health issue and at least one mental health issue.

Dependent variables (caregivers’ physical and mental health problems) and independent variables (caregivers’ characteristics such as age, sex, presence of substitute caregivers, and use of Long-Term Care Insurance System and care recipients’ dementia type and number of BPSDs) were analyzed using logistic regression. First, crude odds ratios (ORs) for physical and mental health problems were calculated for each variable (Model 1). Next, all variables were adjusted, and ORs with 95% confidence intervals (CIs) for physical and mental health problems were calculated (Model 2).

All data were analyzed using SPSS version 29.0 (IBM, Armonk, NY, USA), with a significant level of 5%.

### Ethical considerations

This study obtained approval from the Ethics Review Committee of the University of Toyama (approval number: 2015071). All participants provided informed consent during prior visits or telephone contacts with public health nurses.

## Results

The analysis included 75 caregivers living with older adults with dementia. Table 1 lists the descriptive statistics for the participants. Caregivers aged 60-79 years accounted for 56.0% of the sample. Approximately 75% of caregivers were female. Furthermore, 74.7% had substitute caregivers available, while 8% used long-term care insurance services. At least one physical health issue and at least one mental health issue were experienced by 40% and 37.3% of caregivers, respectively. Lower back pain was the most reported physical symptom (18.7%), followed by joint pain in the hands and feet (14.7%). Among mental health problems, irritability (24.0%) was the most prevalent, followed by insomnia (13.3%) and depression (8.0%). Regarding care recipients, 64% were diagnosed with Alzheimer’s disease and 12% with vascular dementia. The most common BPSD was apathy (22.7%), followed by abnormal behavior (21.3%), agitation (20%), and irritability (18.7%). Regarding the number of BPSDs, 54.7% had at least one BPSD, while 45.3% had none.

**Table 1.** Participant Characteristics (n = 75).

Caregivers		n	%
Age (years)	<60	18	24.0
	60-69	29	38.7
	70-79	13	17.3
	≥80	15	20.0
Sex	Male	18	24.0
	Female	57	76.0
Caregiver substitute	Yes	56	74.7
	No	19	25.0
Use of Long-Term Care Insurance System	Yes	6	8.0
	No	69	92.0
Physical health problems	At least one symptom	30	40.0
	Lower back pain	14	18.7
	Joint pain in the hands and feet	11	14.7
	Insufficient sleep	8	10.7
	Stiff shoulders	8	10.7
	Generalized fatigue	7	9.3
	Other	8	10.7
Mental health problems	At least one symptom	28	37.3
	Irritation	18	24.0
	Insomnia	10	13.3
	Depression	6	8.0
	Other	3	4.0
Care recipients		n	%
Dementia types	Alzheimer’s disease	48	64.0
	Vascular dementia	9	12.0
	Mixed dementia	6	8.0
	Lewy body dementia	4	5.3
	Other	8	10.7
BPSDs	Apathy	17	22.7
	Abnormal behavior	16	21.3
	Agitation	15	20.0
	Irritability	14	18.7
	Delusion	10	13.3
	Hallucination	8	10.7
	Anxiety	8	10.7
	Depression	6	8.0
Total number of BPSDs	At least one	41	54.7
	0	34	45.3
	1	14	18.7
	≥2	27	36.0

BPSD: behavioral and psychological symptom of dementia.

Table 2 shows the association between physical health problems among caregivers and independent variables, as well as the proportions of caregivers with physical health problems for each independent variable. In Model 1, which calculated crude ORs, caregivers’ age and sex showed significantly higher ORs. In Model 2, which adjusted for all variables, only caregivers’ age exhibited a significantly higher OR (19.74, 95% CI 2.78-140.04 for caregivers aged ≥80 years vs. the reference group aged <60 years). Although not significant, physical health problems were more common among male caregivers than among female caregivers. The dementia type of care recipients, the number of BPSDs, the presence of substitute caregivers, and the use of the Long-Term Care Insurance System demonstrated no association with caregivers’ physical health problems in Model 2.

**Table 2.** Factors Associated with Caregivers’ Physical Health Problems.

		Caregivers’ physical health problems, yes	Model 1			Model 2		
		%	OR	95% CI	p-Value	OR	95% CI	p-Value
Caregivers’ age (years)	<60	16.7	1.00			1.00
	60-69	34.5	2.63	0.61-11.30	0.193	2.73	0.57-13.04	0.208
	70-79	38.5	3.13	0.59-16.58	0.181	3.70	0.61-22.43	0.154
	≥80	80.0	20.00	3.40-117.56	<0.001	19.74	2.78-140.04	0.003
Caregivers’ sex	Male	61.1	3.14	1.05-9.40	0.041	2.80	0.73-10.74	0.134
	Female	33.3	1.00			1.00		
Care recipients’ dementia type	Alzheimer’s type	39.6	1.00			1.00		
	Cerebrovascular	44.4	1.22	0.29-5.14	0.785	1.43	0.28-7.48	0.669
	Mixed type	50.0	1.53	0.28-8.37	0.626	1.08	0.11-10.34	0.948
	Lewy body dementia	50.0	1.53	0.20-11.78	0.685	1.12	0.09-13.57	0.929
	Other	25.0	0.51	0.09-2.79	0.436	0.82	0.13-5.18	0.833
Care recipients’ total number of BPSDs	0	38.2	1.00			1.00		
	1	28.6	0.65	0.17-2.49	0.526	0.80	0.16-3.93	0.779
	≥2	48.1	1.50	0.54-4.18	0.438	1.12	0.31-4.03	0.867
Caregiver substitute	Yes	41.1	1.00			1.00		
	No	36.8	0.84	0.29-2.45	0.745	1.16	0.31-4.30	0.829
Use of long-term care insurance services	Yes	50.0	1.00			1.00		
	No	39.1	0.64	0.12-3.42	0.604	1.37	0.14-13.61	0.788

Notes: Model 1 included crude ORs; Model 2 was adjusted for caregivers’ age, caregivers’ sex, care recipients’ dementia type, care recipients’ total number of BPSDs, caregiver substitute, and use of the Long-Term Care Insurance System. BPSD: behavioral and psychological symptom of dementia; CI: confidence interval; OR: odds ratio.

Table 3 shows the association between caregivers’ mental health problems and the independent variables, as well as the proportions of caregivers with mental health problems for each independent variable. In Model 1, the OR increased as the number of BPSDs of the care recipients increased, becoming significantly higher for two or more BPSDs compared with no BPSDs. In Model 2, which adjusted for all covariates, the association between having two or more BPSDs and caregivers’ mental health problems was attenuated, although a marginal association remained (adjusted OR 2.96, 95% CI 0.84-10.43; p = 0.091). Unlike physical health problems, mental health problems had no significant association with caregivers’ age. Although not significant, male caregivers had a higher proportion of mental health problems than female caregivers. Similarly, caregivers aged under 60 years reported more mental health problems than other age groups, and caregivers of older adults with Lewy body dementia reported more mental health problems than those of older adults with other dementia types.

**Table 3.** Factors Associated with Caregivers’ Mental Health Problems.

		Caregivers’ mental health problems, yes	Model 1			Model 2		
		%	OR	95% CI	p-Value	OR	95% CI	p-Value
Caregivers’ age (years)	<60	38.9	1.00			1.00		
	60-69	31.0	0.71	0.21-2.42	0.581	0.47	0.12-1.91	0.294
	70-79	30.8	0.70	0.15-3.17	0.642	0.84	0.16-4.51	0.834
	≥80	53.3	1.80	0.45-7.20	0.408	1.28	0.25-6.62	0.765
Caregivers’ sex	Male	50.0	2.00	0.68-5.86	0.207	1.79	0.94-6.49	0.375
	Female	33.3	1.00			1.00		
Care recipients’ dementia type	Alzheimer’s type	35.4	1.00			1.00		
	Cerebrovascular	33.3	0.91	0.20-4.11	0.904	0.86	0.16-4.65	0.864
	Mixed type	50.0	1.82	0.33-10.04	0.490	1.74	0.24-12.88	0.586
	Lewy body dementia	75.0	5.47	0.53-56.75	0.154	5.76	0.41-80.43	0.193
	Other	25.0	0.61	0.11-3.35	0.567	1.16	0.18-7.59	0.876
Care recipients’ total number of BPSDs	0	23.5	1.00			1.00		
	1	42.9	2.44	0.65-9.15	0.187	3.08	0.72-13.19	0.130
	≥2	51.9	3.50	1.17-10.46	0.025	2.96	0.84-10.43	0.091
Caregiver substitute	Yes	37.5	1.00			1.00		
	No	36.8	0.97	0.33-2.86	0.959	1.33	0.38-4.65	0.661
Use of Long-Term Care Insurance System	Yes	50.0	1.00			1.00		
	No	36.2	0.57	0.11-3.03	0.508	0.41	0.06-2.82	0.365

Notes: Model 1 included crude ORs; Model 2 was adjusted for caregivers’ age, caregivers’ sex, care recipients’ dementia type, care recipients’ total number of BPSDs, caregiver substitute, and use of the Long-Term Care Insurance System. BPSD: behavioral and psychological symptom of dementia; CI: confidence interval; OR: odds ratio.

Additional exploratory analyses were conducted examining each physical and mental symptom separately as outcome variables using logistic regression models including all independent variables. Among physical symptoms, sleep deprivation was significantly more common in male caregivers than in female caregivers (adjusted OR 15.87, 95% CI 1.06-238.34; p = 0.046). No statistically significant associations were observed for mental symptoms (p >0.05).

## Discussion

This study demonstrated the prevalence of physical and mental health problems among caregivers of older adults with dementia. Approximately 40% of all caregivers included in this study experienced physical or mental health problems. Furthermore, 64% of care recipients had at least one BPSD. Caregivers’ age was associated with physical health problems, while the number of BPSDs showed a marginal association with mental health problems.

This study has two strengths. First, participants were randomly selected from all residents aged 65 years or older living in Toyama prefecture, significantly reducing selection bias compared with other studies conducted in clinical settings. Second, teams consisting of a psychiatrist and a public health nurse visited all participants at home to diagnose dementia, enabling the exclusion of older adults without dementia. While population-based studies using questionnaires or interviews without psychiatrists are common, this study achieved high validity by involving psychiatrists.

In this study, 40% of family caregivers experienced physical health problems, while 37.3% experienced mental health problems. Previous studies tended to show that numerous caregivers manifested some symptoms. Onishi et al. ^(10)^ reported that 79.3% of caregivers experienced some form of physical pain, while Koyama et al. ^(25)^ stated that approximately 39% of caregivers aged under 65 years suffered from sleep disorders. Another study also reported depression in 32% of caregivers ^(26)^. The discrepancy of results between our study and these previous studies is likely attributable to differences in study design (whether hospital based or community based). Caregivers accompanying family members with dementia to medical appointments may report more health problems than those who do not accompany them.

This study found a significant association between caregivers’ age and physical health problems. The higher the age of the caregiver, the greater the proportion experiencing physical health problems. In particular, caregivers’ age of 80 years or older had a significant association with physical health problems. There was no statistically significant relationship between mental health problems and age. Caregivers aged 50 years or younger reported more mental health problems than those aged 60-79 years, with ORs showing a U-shaped pattern across the three age groups. The reason younger caregivers tend to experience poorer health likely relates to societal demands associated with different roles and expectations.

Previous studies have reported on the relationship between caregivers’ age and caregiving burden; however, the results were inconsistent. Lim et al. ^(27)^ reported a positive correlation between caregivers’ burden and age, while Onishi et al. ^(10)^ found a negative correlation. Meanwhile, Paddick et al. ^(28)^ showed no correlation between caregivers’ age and burden. In these studies, age was considered a continuous variable. This may explain the lack of consistency. Significant differences in U-shaped results are difficult to demonstrate using continuous variables. In our study, age was treated as a categorical variable; thus, a marked trend was noted in its association with physical health problems. Furthermore, the prevalence of mental health problems was lower in our study than in previous studies using care burden scales such as the ZBI or SF-8 as dependent variables. This result may stem from the fact that mental health problems reflect more severe circumstances than care burden, potentially leading to its underreporting.

According to previous international studies, female caregivers experience greater burden than male caregivers ^(13)^. However, this study showed an opposite trend. Male caregivers tended to experience more physical and mental health problems than female caregivers, although this association was not statistically significant. In Japan, Koyama et al. ^(25)^ reported lower SF-8 scores among male caregivers than among female caregivers. Cultural differences or the tendency of Japanese male caregivers to be reluctant to consult healthcare providers or staff may explain this finding. Further research focusing on sex differences in Japan is needed.

Moreover, at least one BPSD was found in 54.7% of care recipients with dementia. Previous studies using the NPI scale reported that the BPSD prevalence rate was 84%-93% ^(13), (28), (29)^, which is higher than that in our study. This discrepancy may be attributable to differences in survey methodology. Specifically, differences exist between interviews conducted by psychiatrists and nurses in hospital settings and those in community settings, where participants often have low hospital visitation rates and may be reluctant to report symptoms to healthcare providers or even to their families. These differences may have contributed to the disparity in BPSD prevalence rates.

In addition, having a caregiver substitute or using Japan’s Long-Term Care Insurance System showed no association with physical or mental health problems among caregivers. One possible factor is that only <10% of the participants used the Long-Term Care Insurance System, indicating a small sample size. Approximately 75% of the caregivers reported having a caregiver substitute, suggesting that they may not need such insurance services; meanwhile, roughly 25% were providing care without a substitute. The fact that many caregivers in the community, even those caring for individuals diagnosed with dementia by a physician, do not or cannot access the Long-Term Care Insurance System is serious. Thus, the entire community must be mindful of caregivers overwhelmed by daily caregiving and must establish systems to support them.

Past studies have reported that dementia type and BPSD correlate with caregiver burden ^(10), (11), (12), (28)^. Additionally, BPSD has been associated with mental disorders among caregivers ^(25), (26)^. These results are similar to those of our study, which showed that the number of BPSDs was marginally associated with caregivers’ mental health problems (Table 3, Model 2). Therefore, all individuals caring for older adults with dementia must adopt strategies that prevent care recipients from developing BPSD, even after dementia develops. Furthermore, although not statistically significant, Lewy body dementia was associated with a higher proportion of caregivers experiencing mental health problems compared with other dementia types. Previous studies have also reported greater caregiving burden among caregivers of older adults with Lewy body dementia ^(12)^. Therefore, healthcare staff should recognize the distinct factors contributing to caregivers’ physical and mental health problems and provide support aimed at preventing the worsening of both.

In the exploratory analyses examining each symptom separately, sleep deprivation was significantly more common among male caregivers than among female caregivers. This finding suggests that male caregivers may be more vulnerable to sleep problems. One possible explanation is that men traditionally have less experience with caregiving and household tasks, which may make adaptation to the caregiving role more challenging. In addition, coping strategies for caregiving stress may differ by sex, potentially contributing to sleep disturbances among male caregivers. In contrast, no significant associations were observed for mental health symptoms. Previous studies have suggested that men may be less likely to report psychological distress and may instead experience stress in the form of physical symptoms. However, as this was an exploratory analysis and the CIs were wide, these findings should be interpreted with caution. Further studies with larger sample sizes are needed to clarify sex differences in sleep problems among caregivers.

This study has several limitations. First, although it was a cross-sectional survey of a random sample of all older adults residing in Toyama prefecture, the number of caregivers analyzed was relatively small (n = 75). However, the Toyama Dementia Survey employed random sampling of older adults residing in Toyama prefecture, and in the second-stage survey, psychiatrists and public health nurses conducted home visits to participants. Therefore, the influence of socioeconomic and geographic factors within Toyama prefecture is likely to be minimal. Accordingly, the findings of this study may have a relatively high degree of generalizability and may also be relevant to other regions. Second, information on caregivers’ preexisting health conditions was not collected. Therefore, we were unable to adjust for this potential confounder in the multivariable analysis. Preexisting health conditions may have influenced caregivers’ physical and mental health outcomes, and residual confounding cannot be excluded. Third, the items used to measure physical and mental health problems as outcomes were not standardized. Fourth, although we used a unique checklist for BPSD rather than the NPI, it lacked two items, namely, “euphoria” and “disinhibition,” compared with the NPI. Nonetheless, no studies have reported a strong association between these two missing factors and caregiver burden ^(13), (29)^. Therefore, our findings regarding the association between the number of BPSD items and caregivers’ mental health problems are considered consistent with other studies using the NPI. Finally, given its cross-sectional design, this study cannot infer the direction of causality between health problems and their factors. Larger-scale longitudinal studies or intervention studies must be conducted to clarify causal relationships and promote caregiver health maintenance.

In conclusion, this study demonstrated a high prevalence of physical and mental health problems among caregivers of older adults with dementia living at home. Caregivers’ physical health problems were associated with their age, while mental health problems were marginally associated with the number of BPSDs exhibited by the care recipients. Given these findings, healthcare professionals managing older adults with dementia need to pay attention to various factors related to caregivers’ health status. In Japan, providing outreach and health programs that aim to maintain caregivers’ health and detect caregivers’ health problems early is desirable.

## Article Information

### Acknowledgments

The authors express their sincere appreciation for the cooperation of the Toyama Prefectural Health and Welfare Bureau, Toyama Neuropsychiatrists Association, the contributing members of the Toyama municipalities, and all survey participants.

### Author Contributions

Nobue Nakahori, Masaaki Yamada, Michikazu Sekine, and Takashi Tatsuse contributed to the study design; data acquisition, analysis, and interpretation; and manuscript preparation. All authors read and approved the final manuscript.

### Conflicts of Interest

None

### Ethics Approval

This study was approved by the Ethics Review Committee of the University of Toyama (approval number: R2015071).
